# Advances and Future Trends in Nanozyme-Based SERS Sensors for Food Safety, Environmental and Biomedical Applications

**DOI:** 10.3390/ijms26020709

**Published:** 2025-01-15

**Authors:** Xingyu Wang, Xuemei Tang, Chengzhen Ji, Long Wu, Yongheng Zhu

**Affiliations:** 1 College of Food Science and Technology, Shanghai Ocean University, Shanghai 201306, China; 18616302670@163.com; 2 Key Laboratory of Tropical Fruits and Vegetables Quality and Safety for State Market Regulation, School of Food Science and Engineering, Hainan University, Haikou 570228, China; 3 State Key Laboratory of Marine Food Processing and Safety Control, Dalian Polytechnic University, Dalian 116034, China

**Keywords:** nanozyme, food safety, surface modification, environmental monitoring, biomedical diagnostics, enzyme-mimicking activities

## Abstract

Nanozymes, a kind of nanoparticles with enzyme-mimicking activities, have attracted considerable attention due to their robust catalytic properties, ease of preparation, and resistance to harsh conditions. By combining nanozymes with surface-enhanced Raman spectroscopy (SERS) technology, highly sensitive and selective sensors have been developed. These sensors are capable of detecting a wide range of analytes, such as foodborne toxins, environmental pollutants, and biomedical markers. This review provides an overview of recent advancements in the synthesis and surface modification of nanozymes, highlighting their ability to mimic multiple enzymes and enhance catalytic performance. In addition, we explore the development and applications of nanozyme-based SERS sensors in food contaminants, environmental pollutants, and biomedical markers. The review concludes with perspectives and challenges facing the field, involving the need for deeper understanding of nanozyme principles and mechanisms, development of standardized systems for characterization, and the engineering of nanozymes with tailored properties for specific applications. Finally, we discuss the potential for integrating various techniques with nanozymes to create multi-modal detection platforms, paving the way for the next generation of analytical tools in the fields of food safety, environmental monitoring, and biomedical diagnostics.

## 1. Introduction

Nanomaterials, renowned for their distinct mechanical, electrical, magnetic, optical, and catalytic properties, have become a focal point across diverse sectors, including electronics [1], healthcare [2], aerospace [3], environmental science [4], energy [5], and biotechnology [6]. As nanotechnology progresses, a plethora of nanomaterials with specialized characteristics has been synthesized and investigated, among which nanozymes—particles that mimic enzymatic activity—have emerged as a significant area of interest [7,8]. Notably, since the discovery in 2007 that Fe_3_O_4_ nanoparticles exhibit peroxidase-like activity akin to horseradish peroxidase (HRP) without the need for surface modification, nanozymes have garnered extensive research attention [9,10]. In the presence of H_2_O_2_, these nanoparticles are capable of catalyzing the oxidation of o-Phenylenediamine (OPD), and 3,3′,5,5′-tetramethylbenzidine (TMB), mirroring the color change induced by HRP.

Traditional sensing methods, such as colorimetric, electrochemical, and fluorescence-based assays, have been widely used for detecting various analytes. However, these methods often face several limitations. For instance, colorimetric assays can suffer from low sensitivity and specificity, making them inadequate for detecting trace amounts of analytes in complex matrices. Electrochemical methods, while offering good sensitivity, can be limited by the need for complex instrumentation and the challenge of achieving stable electrode surfaces. Fluorescence-based assays, although highly sensitive, are prone to photobleaching and interference from background fluorescence, which can lead to false-positive results. To address these challenges, the integration of nanozymes with surface-enhanced Raman spectroscopy (SERS) technology has led to the development of innovative sensing platforms. SERS, a powerful vibrational spectroscopy technique, provides molecular fingerprinting capabilities with high spatial resolution, making it an ideal platform for enhancing the performance of nanozyme-based sensors. The nanoscale roughness or plasmonic properties of SERS-active substrates amplify the Raman signals of molecules in close proximity, thus enabling the detection of even single molecules.

Nanozymes have indeed demonstrated extensive utility in in vitro detection and within living systems, presenting themselves as potential direct replacements for natural enzymes, particularly in the context of advanced analytical techniques [11]. They offer several advantages over natural enzymes, such as ease of modification and purification, which are critical for tailoring their properties to suit specific applications [12]. The size, shape (e.g., nanospheres, nanosheets, nanorods, nanowires), and surface chemistry of nanozymes significantly influence their enzymatic activity, enabling their versatile use in developing analytical methods. [13]. These characteristics allow nanozymes to be designed with controllable activity and resistance to environmental conditions, making them strong competitors to natural enzymes.

Owing to their high catalytic efficiency, low cost, and robustness against harsh environmental conditions, nanozymes have been instrumental in revolutionizing the field of biosensing [14]. Their unique benefits include adjustable catalytic activity, high stability under extreme conditions (e.g., high temperature, oxidative stress, extreme pH level), flexibility in composition and structural design, and excellent biocompatibility, making them ideal candidates for mimicking enzymes. When integrated with traditional optical, electrochemical, or colorimetric assays, nanozymes can enhance the sensitivity and stability of analytical methods [15,16]. Especially, the combination of nanozymes with SERS sensors significantly improves detection limits, allowing for the identification of trace amounts of target molecules with exceptional performance [17,18,19]. The synergy not only boosts analytical performance but also broadens the range of applications, making these sensors suitable for on-site, real-time monitoring in food safety, environmental pollution detection, and biomedical marker identification.

**Figure 1:**
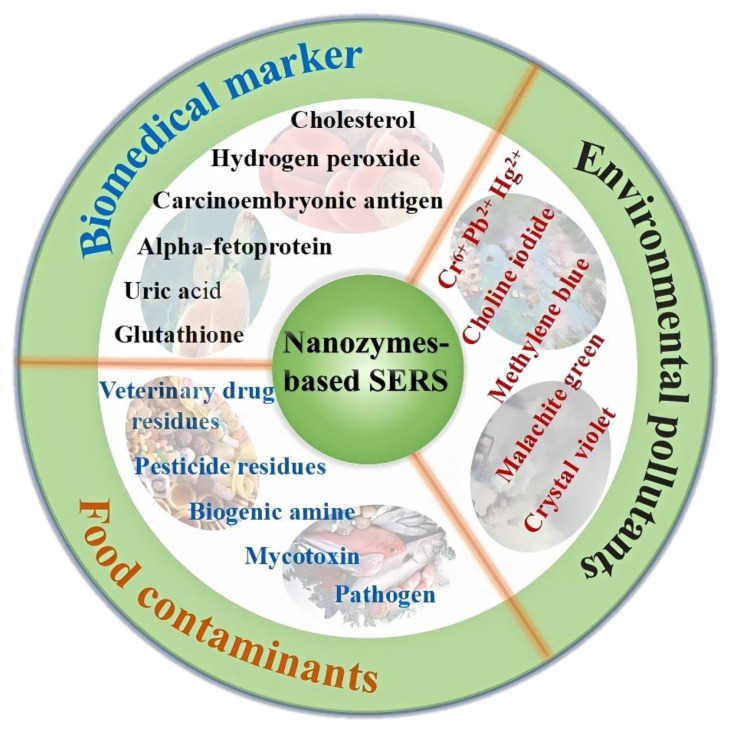
Schematic presentation of nanozymes-based SERS sensors for the detection of food contaminants, environmental pollutants, and biomedical markers.

In this review, we will explore the recent advancements in the synthesis and surface modification of nanozymes, their multienzyme-like activities, and the enhancement of catalytic performance (Figure 1). Furthermore, we will discuss the applications of nanozyme-based SERS sensors in detecting various contaminants and markers and conclude with perspectives on the challenges and future trends in the field, including the development of multi-modal detection platforms that integrate distinct techniques with nanozymes.

## 2. Synthesis and Surface Modification of Nanozymes

### 2.1. Synthesis of Nanozymes

Recent advancements in the synthesis of nanozymes mainly include the exploration of different nanozymes, such as metal–organic frameworks (MOFs), single-atomic and dual-atomic-site catalysts, manganese-based nanozymes, and regulation of their catalytic activity [20]. Innovations in nanozyme synthesis have significantly enhanced the development and application of these artificial enzymes, making them more versatile for various uses [21]. For example, recent studies have highlighted the use of MOFs as templates or precursors for synthesizing nanozymes [22]. This approach exploits the advantages of MOFs while addressing their inherent limitations, such as agglomeration and limited hydrophilicity, which can impair catalytic performance. The classification of MOF-based nanozymes has progressed, with research now focusing on three main types: pure MOF-based nanozymes [23], metal-doped variants [24], and functionalized versions [25]. These advancements aim to enhance catalytic efficiency and broaden the practical applications of nanozymes in biotechnology.

Furthermore, the exploration of single-atomic nanozymes has gained traction due to their well-defined spatial configurations and versatile enzyme-like performances [26]. More recently, dual-atomic-site catalysts (DACs) have emerged as a promising direction for nanozyme design [27]. DACs, which consist of two adjacent single-atomic sites, offer greater flexibility in adjusting active sites and enhancing catalytic activity [28]. This innovation marks a significant step forward in the synthesis of nanozymes with enhanced functionalities.

Additionally, manganese oxide nanoparticles have been found to possess intrinsic enzyme-like activities, including peroxidase, glutathione peroxidase, catalase, and superoxide dismutase [29]. Recent advancements have focused on the synthesis of manganese-doped nanoparticles that enhance therapeutic potential, particularly in tumor therapy by generating reactive oxygen species in response to specific stimuli in the tumor microenvironment [30].

New techniques have been developed to control the activity of nanozymes using external triggers [31]. For instance, it has been demonstrated that visible light can adjust the antibacterial properties of certain nanozymes, like copper oxide (CuO) nanorods [32]. These nanorods show peroxidase-like behavior that can be managed by exposure to light. This ability to fine-tune enzyme-like activities in response to environmental changes opens new avenues for targeted therapeutic applications [33]. In addition, the synthesis of nanozymes has also expanded to include various materials such as noble metals, transition metal oxides, and carbon-based nanomaterials [34]. This variety has contributed to the development of nanozymes with specific functionalities tailored for applications in biosensing, biomedicine, food safety, and environmental protection.

### 2.2. Techniques for Surface Modification

For surface modification of nanozymes, it is a critical technique that significantly influences their catalytic activity, stability, and overall performance in multiple applications [35]. Numerous studies have underscored the pivotal role of structural properties, such as size, morphology, and surface groups, in dictating the catalytic prowess of nanozymes [36]. Generally speaking, as the size diminishes, their specific surface area expands, leading to a pronounced increase in the number of unsaturated coordination sites on the surface atoms [37]. Consequently, this surge in surface active sites bolsters the catalytic efficiency, implying that smaller nanozymes tend to exhibit heightened catalytic activity [38]. In this context, the enzyme-like activity can be fine-tuned by meticulously controlling their size.

Furthermore, the morphology and interface structure of nanozymes are susceptible to alterations under varying reaction conditions, which in turn can significantly impact their catalytic performance [39]. By strategically exposing the crystal facets with the highest activity or specific energy, one can substantially enhance the catalytic activity of nanoparticles [40]. The morphology-dependent behavior of nanozymes can be attributed to the distinct lattice arrangements of atoms within different structural forms, which result in varying surface activities and catalytic performances [40]. This understanding allows for the design of nanozymes with tailored properties to meet specific catalytic demands in various applications.

**Figure 2:**
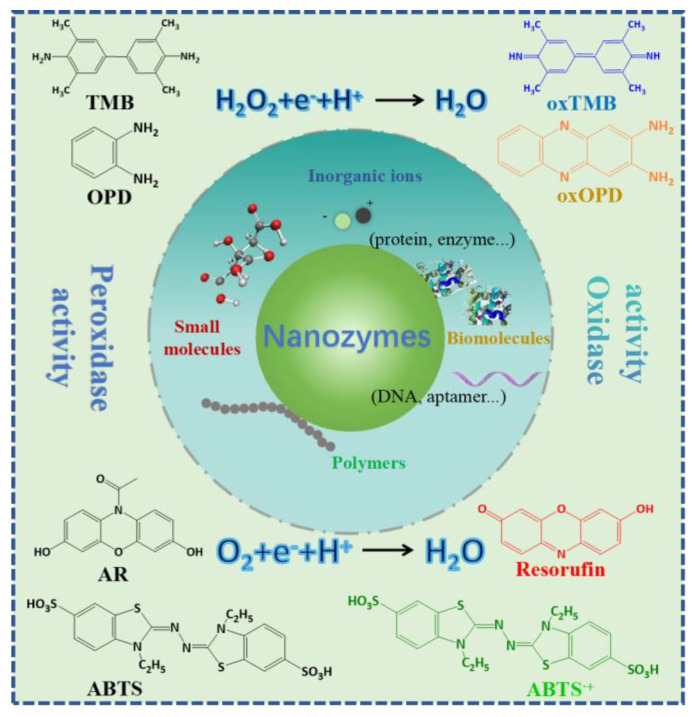
Surface modification of nanozymes with small molecules, biomolecules, polymers, and inorganic ions.

Furthermore, the capabilities of nanozymes can be reshaped and enhanced through a multitude of surface modification techniques, including altering charges, applying coatings, introducing functionalization, and loading additional components (Figure 2) [35]. These strategies enable the achievement of targeted recognition and catalytic activities that are contingent upon the specific surface properties engineered into the nanozymes [41]. Successful examples of such modifications include the incorporation of ions, small molecules, nucleotides and nucleic acids, amino acids and peptides, proteins, and polymers, among others [42]. These modifications not only amplify the functionalities of nanozymes but also broaden their scope of application.

Nanozymes with uniquely engineered surfaces can achieve sensitive and specific recognition and detection of analytes [43]. This precision is crucial in applications such as biosensing, where the ability to selectively identify and respond to specific targets is paramount [44]. The tailored surface properties facilitate the binding of the nanozymes to their intended targets, leading to enhanced catalytic activities that are dependent on the presence of these targets [45]. This surface engineering approach thus provides a versatile platform for the development of sensitive and selective detection systems, capable of addressing assorted analytical challenges in fields such as environmental monitoring, biomedical diagnostics, and food safety.

### 2.3. Catalytic Performance and Multienzyme-Like Activities

Nanozymes, as a distinct category of artificial enzymes, offer a range of desirable functions that extend beyond mere catalytic activity [46]. Their synthesis can be derived from a diverse array of sources, ranging from metals to metal oxides and carbon materials, which ensures their ready availability [47]. The use of inorganic raw materials and the mild conditions under which most nanozymes are prepared contribute to their unique attributes, such as low cost, ease of storage, resistance to denaturation, and high stability [48]. The characteristics make nanozymes particularly attractive for a variety of applications.

Moreover, many nanozymes possess the ability to mimic the functions of multiple natural enzymes, exhibiting multienzyme-like activities [49]. For instance, depending on the pH conditions, nanoparticles such as CeO_2_ and Au can display activities akin to superoxide dismutase, peroxidase, oxidase, and catalase [50]. The catalytic performance of these nanozymes is often assessed through kinetic characterization, with Michaelis−Menten kinetics experiments being a standard approach to compare their activity with that of natural enzymes. This method allows for the establishment of unified standards in terms of substrate specificity (Km), catalytic rate constant (kcat), and catalytic efficiency (kcat/km).

In general, nanozymes are known to mimic the activities of oxidases, hydrolases, superoxide dismutases, and catalases [51]. The catalytic mechanisms and kinetics of these nanozymes have been extensively discussed under various conditions, such as pH, temperature, and dissolved oxygen levels [52]. However, as mentioned, a single type of nanomaterial can exhibit several different functions, which may be pH-dependent or related to structural properties like size, morphology, surface groups, and defects [53]. The intricate interplay between physicochemical properties and catalytic characteristics necessitates a systematic guide for the engineering and design of nanozymes. This guide would facilitate the development of nanozymes tailored to specific applications, optimizing their performance and ensuring their reliability in diverse environments.

## 3. Nanozyme-Based SERS Sensors

### 3.1. Principles of SERS and Nanozyme Interaction

SERS is a powerful analytical technique that amplifies the Raman response of an analyte when it interacts with the surface plasmon of metals such as gold (Au), silver (Ag), or copper (Cu) [54]. This enhancement can be significant enough to achieve single-molecule detection [55]. The exact mechanism behind the signal enhancement in SERS is still a subject of debate, but it is generally accepted to be driven by two main principles: the electromagnetic effect and the chemical effect.

The EM mechanism is the most understood aspect of SERS [56]. It originates from the substrate and occurs when a free-electron-like metal is irradiated with a laser whose frequency is resonant with the collective oscillation of conduction band electrons, a phenomenon known as surface plasmon resonance (SPR) [57]. In regions called hot spots, an intense local field enhancement is produced around the metal interface by the concentration of light, creating an oscillating dipole on molecules in close proximity to the nanoparticles with an enhanced radiation efficiency [58]. The chemical effect involves charge transfer between the metal and the adsorbed molecules, leading to an enhancement of the Raman signal. This effect is generally considered to be of lesser magnitude compared to the EM effect but can contribute significantly under certain conditions.

Nanozymes, with their enzyme-mimicking activities, can be integrated with SERS to create highly sensitive and selective sensors [59]. Firstly, nanozymes can catalyze the conversion of substrates to oxidized coloring products, similar to natural enzymes [60]. This catalytic activity can be coupled with SERS to provide a signal that is proportional to the concentration of the analyte [61]. The SERS-active nanozymes can generate a unique fingerprint peak, providing more information about the analyte and enhancing the detection sensitivity [62]. Secondly, nanozymes can be designed to possess the dual properties of both nanozymes and SERS substrates [63]. They can be synthesized via top-down or bottom-up strategies and combined with SERS-active ingredients like gold and silver to generate nanozyme SERS substrates [64]. This integration allows for the direct provision of SERS signals without the need for additional substrates when performing SERS applications [65]. Thirdly, SERS provides a novel and powerful method for studying the reaction kinetics of nanozymes [66]. It can precisely monitor changes in adsorbed molecules on the catalyst surface as well as the catalytic process, offering a distinct advantage over conventional colorimetric methods that can only reflect the reaction process in solution and not at the catalyst interface.

The integration of nanozymes with SERS principles enhances the detection limits by several orders of magnitude, allowing for the identification of trace amounts of target molecules with unparalleled precision. This synergy between nanozymes and SERS has revolutionized the field of biosensing, providing analytical tools that are both powerful and precise.

### 3.2. Design and Fabrication of Nanozyme-Based SERS Sensors

Under the technique integration of nanozymes and SERS detection, the design and fabrication of nanozyme-based SERS sensors involve several critical steps, mainly including nanozyme selection and synthesis, integration with SERS-active substrates, surface modification, signal amplification strategy, and dual-mode detection [67,68]. Coupled with the properties of nanozymes, the design of nanozyme-based SERS sensors is often tailored to specific applications, such as disease diagnosis, food safety, and environmental monitoring.

To be specific, the main step for the design of nanozyme-based SERS sensors is the selection and synthesis of appropriate nanozymes that can be derived from various nanomaterials, including gold, silver, platinum, molybdenum sulfide, zeolites, and more [69,70]. Compared to natural enzymes, these materials possess enzyme-like catalytic activity and offer advantages such as low cost, simple preparation methods, robust catalytic activity, smooth surface modification, and high stability [67]. With the prepared nanozymes, they are integrated with SERS-active substrates, typically noble metals like gold or silver, which provide the surface plasmon resonance necessary for signal enhancement [71]. The integration can be achieved by growing nanoparticles in situ on support materials or by modifying the surface of nanozymes with SERS-active ingredients [72]. To enhance their catalytic activity, the nanozyme surfaces are often modified to attach specific recognition elements, such as antibodies or aptamers, for target recognition. This modification can improve the stability of the nanozymes and their interaction with the SERS substrate.

In the design of SERS sensors, nanozymes also serve as labels for multi-category signal amplification [73]. They can catalyze the conversion of substrates to produce a detectable signal that is enhanced by SERS substrates [74]. This signal amplification is crucial for achieving the high sensitivity required for detecting trace amounts of analytes [75]. The fabrication of SERS substrates involves controlling the stability of nanostructures and plasmonic effects around hot spots to limit fluctuations in SERS signals [76]. Techniques such as electron beam lithography, nanowire templating, and nanosphere lithography are used to create SERS substrates with precise adjustable hot spots [77]. Additionally, some nanozyme-based SERS sensors are designed to operate in dual modes, providing both colorimetric and SERS detection capabilities [78]. This dual-mode assay can offer color changes and SERS intensity directly correlating to analyte concentrations, enhancing the reliability and versatility of the sensor.

In a word, the design and fabrication of nanozyme-based SERS sensors involve a combination of material selection, surface modification, SERS substrate development, and application-specific optimization to create highly sensitive and selective detection platforms.

## 4. Application of Nanozyme-Based SERS Sensors

Nanozyme-based SERS sensors leverage the catalytic properties of nanozymes and the signal enhancement capabilities of SERS to create highly sensitive and selective detection platforms [67]. These sensors are finding increasing application in various fields, including food safety, where they detect contaminants like toxins and pesticides; environmental monitoring for the identification of pollutants and heavy metals [79,80,81]; and biomedical diagnostics for the early detection of disease markers and therapeutic monitoring [82]. Their ability to provide molecular fingerprinting with high precision makes them invaluable tools for analytical chemistry, offering a robust alternative to traditional enzyme-based sensors due to their stability, ease of synthesis, and resistance to denaturation [59]. The versatility of nanozyme-based SERS sensors is further enhanced by their potential for dual-mode detection and the ability to regulate their properties for specific analytical targets, making them a cutting-edge technology in the realm of analytical science.

### 4.1. Food Contaminants Detection

**Figure 3:**
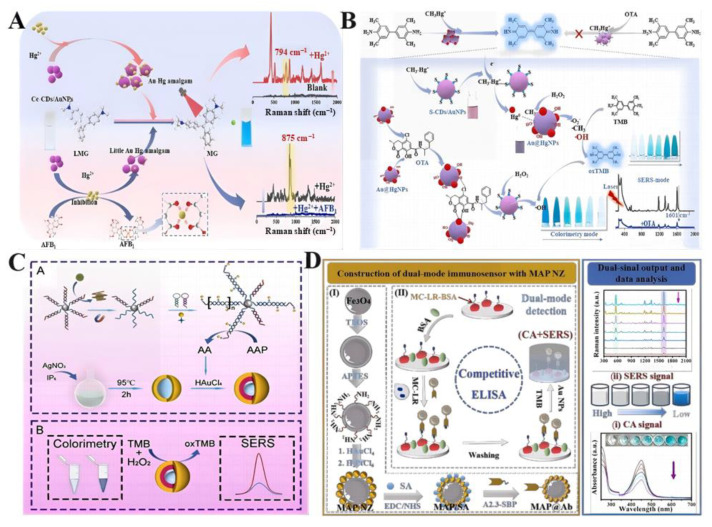
(**A**) Schematic illustration of the detection of Hg^2+^ and AFB1 based on Au@HgNPs [95]. (**B**) Schematic diagram of MeHg^+^-sensitized SERS-active POD-like activity of S-CDs/AuNPs and the “turn off” of OTA [96]. (**C**) Colorimetric/SERS dual-mode detection based on Ag@Au IP6 core–shell nanozyme [93]. (**D**) Schematic illustration of the colorimetric-SERS dual-mode immunosensor for rapid detection of MC-LR based on MAP@Ab probes catalyzed TMB [78].

**Figure 4:**
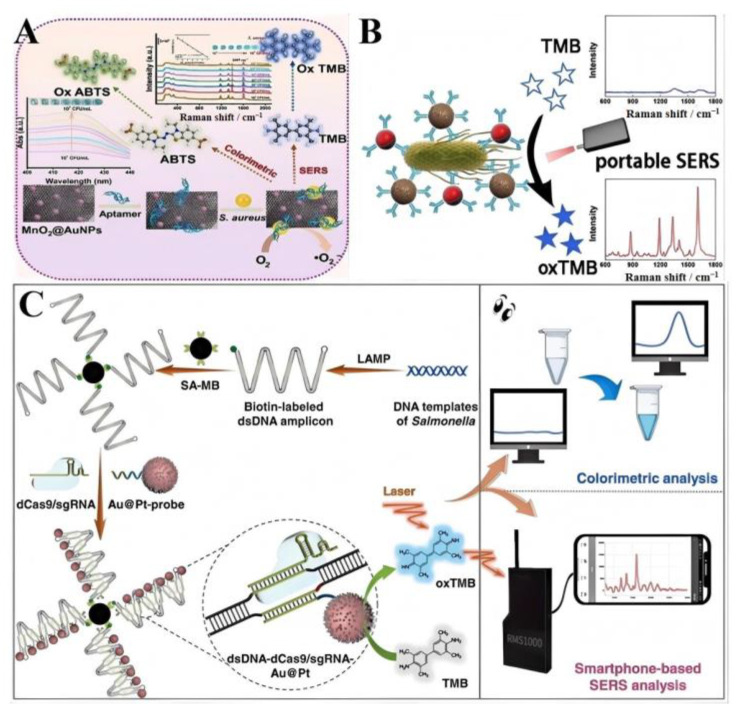
(**A**) Schematic diagram of the synthesis of MnO_2_@AuNPs and the mechanism of specific *S. aureus* detection [101]. (**B**) Illustration for detection of *S. typhi* based on the Au@Pt nanozyme and immunomagnetic beads [102]. (**C**) Scheme of dCas9-CSD for Salmonella assay [10].

Food contaminants are a major health risk and a significant issue for food safety, highlighting the need to develop reliable analytical techniques for monitoring and assessing food safety risks [83,84,85]. In this context, the application of nanozyme-based SERS sensors for detecting food contaminants is a pivotal focus area, given their good sensitivity and specificity [86,87]. Such sensors are being widely adopted for detecting many contaminants, involving mycotoxins (Figure 3), antibiotics, pesticide residues, pathogens (Figure 4), biogenic amine, and others [88,89,90,91,92]. They show numerous benefits over traditional methods, including enhanced selectivity and sensitivity, precise target identification, reduced detection times, and improved signal clarity.

#### 4.1.1. Mycotoxin Detection

For mycotoxin detection using nanozyme-based SERS sensors, Tan et al. present an ultrasensitive dual-mode aptasensor for the detection of aflatoxin B1 (AFB1) in foodstuff (Figure 3C) [93]. The aptasensor utilizes a self-assembled core–shell-structured Ag@Au IP6 bifunctional nanozyme that exhibits peroxidase-like activity, initiating the color reaction and enhancing the SERS signal of oxTMB. The detection strategy combines colorimetric sensing for rapid visual detection and SERS for accurate quantification, achieving a limit of detection (LOD) as low as 0.58 pg/L for AFB1. Based on a peroxidase-like Cu_2_O@Au hybrid nanozyme, Chen et al. describe the development of a SERS aptasensor for detecting AFB1 [94]. The nanozyme produces a synergistic catalytic enhancement effect, and the preferred binding of the aptamer to AFB1 triggers the dissociation of Au-Ag NPs, reducing the SERS effect. The aptasensor shows a strong negative linear correlation with AFB1 concentration and a LOD of 0.7 pg/mL, demonstrating potential in test technology. Furthermore, Zhao reports a novel SERS method for the simultaneous detection of Hg^2+^ and AFB1 using oxidase-like Au@HgNPs/carbon dots (Figure 3A) [95]. AuNPs catalyze the reduction of Hg^2+^ to form Au-Hg amalgam, improving the oxidase-like activity and oxidizing leucomalachite green into Raman-active malachite green. The introduction of AFB1 inhibits the aggregation of Au@HgNPs, reducing the SERS intensity. This work offers a new path for designing nanozyme-based SERS protocols for food analysis.

Li et al. report on the synthesis of gold nanoparticles (S-CDs/AuNPs) with weak peroxidase-like activity that can be selectively and sensitively regulated by methylmercury (MeHg^+^) (Figure 3B) [96]. The catalytic activity of S-CDs/AuNPs is significantly activated by MeHg^+^, leading to an enhancement of the SERS signal. The introduction of ochratoxin A (OTA) turns off both the UV-vis absorbance signals and the SERS signal, establishing a selective colorimetric-SERS dual-mode detection strategy for OTA with low limits of detection. Similarly, Li et al. present a nanozyme-linked apta-sorbent assay (NLASA) for the detection of OTA using Pd-Pt bimetallic nano crystals (Pd-Pt NRs) as nanozyme labels [97]. The NLASA method offers dual-mode detection via colorimetric and SERS methods, with limits of detection of 0.097 nM and 0.042 nM, respectively. The method applied in wine and grape samples shows satisfactory agreement with HPLC-MS/MS results, demonstrating its potential for rapid and sensitive OTA detection in food products. Wu et al. develop a dual-mode immunosensor for the detection of microcystin-LR (MC-LR), a cyanotoxin based on a bifunctional nanobody and Fe_3_O_4_@Au-Pt nanozyme (Figure 3D) [78]. The sensor operates in a microplate with both colorimetric and SERS detection modes with direct correlation between color changes/SERS intensity and MC-LR concentrations. The method eliminates complex enzymatic reactions and enables dual-signal detection of MC-LR in water samples within 30 min.

#### 4.1.2. Pesticide and Veterinary Drug Detection

For pesticide residue detection, Ma et al. propose an indirect SERS sensing assay for the determination of glyphosate (Gly) in tap water [98]. The detection mechanism is based on the relief of the inhibitory effect of L-cysteine (L-cys) on a Au-Pt nanozyme by combining Gly with L-cys through divalent copper ions (Cu^2+^). A novel nanochain-like Au-Ag composite was fabricated for detecting SERS signals of oxTMB without interfering with TMB Raman signals. The assay showed a good linear response over the concentration ranges of 10 μg L^−1^ to 1000 mg L^−1^ with a LOD of 5 μg L^−1^. The method shows good anti-interference ability against interfering cations and structural analogs, making it suitable for practical detection of Gly in tap water. Li et al. describe a new Fe metal–organic framework-loaded liquid crystal (FeMOF@OCTB) nanosol that exhibits good stability and a strong catalytic effect for the polyethylene glycol 400-Ag (I) indicator reaction [99]. The generated Ag NPs have a strong SERS effect and a surface plasmon resonance absorption peak at 420 nm. This new bimodal nanosilver indicator reaction was coupled with isocarbophos (IPS)-aptamer reaction, establishing a SERS/Abs bimodal aptamer assay for IPS. The SERS assay can detect IPS in the concentration range 0.02~1.2 nM, with a LOD of 0.010 nM, and has been applied to the determination of IPS in rice samples.

For veterinary drug detection, Zhang et al. report a dual-mode detection strategy for chloramphenicol (CAP) using magnetic multi-“hotspot” nanoflower particles as SERS substrates and bimetallic peroxidase of Au@Pt [100]. The method integrates an exponential amplification reaction (EXPAR) strategy, achieving a highly sensitive detection of CAP. In the presence of CAP, the EXPAR is activated, generating a substantial amount of amplicons that form a stable “Y-shaped” structure with magnetic nanoparticle probes and nanozyme probes. The addition of TMB results in changes in both color and Raman signals, achieving dual-mode ultra-trace detection of CAP with a SERS detection range from 1.0 × 10^−12^ to 1.0 × 10^−6^ M, and a colorimetric method ranges from 2.5 × 10^−7^ M to 1.0 × 10^−8^ M. Based on Au nanozymes, Li et al. construct an aptamer sensor for the ultrasensitive SERS detection of tobramycin [59]. The tobramycin aptamer adsorbs on the surface of Au NPs, limiting the nanozyme peroxidase activity. The inclusion of tobramycin dislodges the aptamer from the Au NPs surface, restoring the catalytic activity of nanozymes. The restored value of nanozyme activity increases with tobramycin concentration and has a good linear correlation in the concentration range of 10^−10^~10^−1^ M with a LOD of 2.04 × 10^−11^ M. The aptamer biosensors built using the SERS platform offer high sensitivity, precision, and a wide detection range, prospectively employed in food safety and the detection of antibiotic residues.

The above work discusses the innovative application of nanozyme-based SERS sensors for the detection of food contaminants, mainly focusing on glyphosate, isocarbophos, chloramphenicol, and tobramycin, which highlight the development of sensitive, selective, and efficient detection methods that leverage the catalytic properties of nanozymes and the signal enhancement capabilities of SERS. The reported methods demonstrate low LODs, wide linear ranges, and good recovery rates, indicating their potential for practical application in food safety monitoring. The integration of nanozymes with SERS technology not only enhances detection sensitivity but also allows for the discrimination of target contaminants from complex food matrices, offering valuable tools for ensuring food quality and safety.

#### 4.1.3. Pathogen Detection

By combining MnO_2_@AuNPs nanozymes with an aptamer specific to *S. aureus*, Dai et al. explore a dual-mode sensing platform that integrates colorimetric and SERS detection for the identification of Staphylococcus aureus (*S. aureus*) (Figure 4A) [101]. The sensor operates by capturing the bacteria via the aptamer and then uses the MnO_2_@AuNPs to catalyze colorimetric changes and enhance SERS signals. The detection range spans from 10 to 107 CFU mL^−1^, with a LOD of 0.926 CFU mL^−1^ for colorimetric detection and 1.561 CFU mL^−1^ for SERS. The platform demonstrated high recovery rates in real samples, indicating its potential for practical application in food safety monitoring. Li et al. describe the development of a bifunctional Au@Pt core–shell nanozyme with both catalytic and SERS activities for the ultrasensitive detection of Salmonella typhimurium in milk samples (Figure 4B) [102]. The Au@Pt nanozyme amplifies the signal by converting Raman-inactive molecules into Raman-active reporters and serves as an active SERS substrate. The LOD achieved was 10 CFU mL^−1^ with high sensitivity and selectivity. The method was successfully applied to milk samples using a portable Raman spectrometer, highlighting its practical utility in food safety and biosensing. Jiang et al. introduce a dCas9-mediated dual-signal platform that uses loop-mediated isothermal amplification (LAMP) and bifunctional Au@Pt nanozymes for the detection of Salmonella (Figure 4C) [103]. The strategy involves dCas9 for the recognition of amplicons generated by LAM, leading to the assembly of Au@Pt nanozymes into chains that convert TMB into oxTMB, providing both colorimetric and SERS signals. The assay can detect as low as 1 CFU mL^−1^ of Salmonella within 50 min, with good robustness across various real samples, making it a promising tool for pathogen detection.

#### 4.1.4. Biogenic Amine Detection

Biogenic amines, such as histamine, are organic nitrogenous compounds that can be produced from the decarboxylation of histidine by microbial contamination [104]. They are prevalent in various foods, particularly in aquatic products, meat products, and fermented goods [105]. Histamine, in particular, is a significant food safety concern as it can cause allergic reactions and food poisoning when ingested in excessive amounts [106,107,108]. The detection of biogenic amines is crucial for ensuring food safety and preventing health risks associated with their consumption. Nanozymes, which mimic the activity of natural enzymes, have been integrated into SERS sensors to enhance detection capabilities, offering a cost-effective and stable alternative to traditional enzyme-based assays.

Based on MOF/noble metal nanoparticles, Ma et al. establish a sensitive SERS aptamer sensor for the detection of histamine [109]. The method involves the synthesis of MIL-100(Fe) loaded with AuNPs to form MIL-100(Fe)@AuNPs, which is used in the TMB/H_2_O_2_ reaction system. Ag NPs are synthesized to amplify the SERS signal of oxidized TMB. The aptasensor is assembled by functionalizing the nanozyme with nucleic acids and combining it with Ag NPs, resulting in a multifunctional substrate with high catalytic and SERS efficiency. The detection system exploits the specific binding effect of the histamine aptamer to histamine, which induces a decrease in the assembly of Ag NPs on MIL-100(Fe)@AuNPs, leading to a decrease in the SERS signals of oxTMB. The assay demonstrates a linear relationship for histamine detection ranging from 10^−11^ M to 5 × 10^−3^ M with a LOD as low as 3.9 × 10^−12^ M. The recovery ratio in fermented soybean products is between 94.42% and 105.75%, proving its applicability in real samples. This SERS aptasensor provides technical support for food safety during processing and storage.

Based on the bioconjugation of histamine aptamers onto the surface of Au NPs, Wang et al. propose a novel aptamer sensor for the rapid, sensitive, and specific detection of histamine, which enhances the peroxidase activity of the nanozymes [110]. The sensor operates on the principle that the histamine aptamer, when detached from the Au NPs surface upon binding to histamine, leads to a decrease in catalytic activity that can be quantitatively analyzed using the TMB-H_2_O_2_ system. The decrease in enzyme catalytic activity correlates well with increasing histamine concentration, establishing a linear correlation. The constructed histamine aptamer biosensor offers ultrasensitivity, high accuracy, and a wide linear range, with a linear range of 10^−11^~10^−3^ M and a detection limit of 1.22 × 10^−12^ M. This approach can be extended to other schemes where aptamers are combined with Au NPs to detect corresponding molecules, demonstrating the potential for broad application in the detection of biogenic amines. Both sensors exhibit high sensitivity and specificity, with a wide linear detection range and low LOD, making them suitable for real-world food safety applications.

#### 4.1.5. Others

Despite the above food contaminants, nanozyme-based SERS sensors are reported on the detection of allergenic protein and ascorbic acid. For instance, Su et al. exhibit a ratiometric SERS immunosorbent assay utilizing gold nanoparticles doped with covalent organic frameworks (COFs) that exhibit mimic nitroreductase activity for the detection of allergenic proteins [111]. The nanozymes replace traditional enzyme tags in ELISA, offering a cost-effective and stable alternative. The assay introduces 4-nitrothiophenol as a substrate that transforms into 4-aminothiophenol in the presence of NaBH_4_, acting as a bridge to connect gold nanostars and create “hot spots” for SERS signal enhancement. The method demonstrates a LOD of 0.01 ng mL^−1^ for β-lactoglobulin, an allergenic protein, with a wide linear range, showing the potential of this nanozyme-SERS approach for sensitive and specific allergen detection in food safety assessments.

On the basis of GeO_2_@Fe_3_O_4_/Au NPs nanozymes, Qi et al. describe a dual-mode sensing platform that integrates smartphone-readable colorimetry with SERS for the detection of ascorbic acid (AA) [112]. The nanozymes exhibit peroxidase-like activity, enabling the oxidation of colorless TMB into blue TMB oxide. Ascorbic acid inhibits this oxidation, leading to a color fade that is detected by both smartphone imaging and SERS. The platform offers a detection range of 0.5 to 340 μmol L^−1^ and a LOD of 0.6689 μmol L^−1^ for AA, with successful application in fruits, vitamin C beverages, and tablets. This study highlights the potential of nanozyme-based SERS sensors for point-of-care testing and food quality assessment, combining the convenience of smartphone technology with the sensitivity of SERS.

In general, the above application of nanozyme-based SERS sensors in food safety assays is relatively recent, and they have shown great potential in addressing challenges associated with food contamination. Compared to traditional methods, these biosensors exhibit merits such as higher selectivity and sensitivity, more specific target recognition, shorter detection times, and better signal readout. They also overcome the limitations of biological enzymes, such as poor operational stability and high costs associated with preparation, isolation, and purification. Nanozyme-based SERS sensors are emerging as powerful tools in the field of food safety, offering innovative solutions for the rapid, sensitive, and specific detection of a wide range of food contaminants, thereby contributing to the protection of public health and the maintenance of food industry standards.

### 4.2. Environmental Pollutant Detection

The domain of environmental monitoring is currently experiencing a significant rise in the deployment of nanozyme-enhanced SERS sensors, which combine the distinctive attributes of both nanozymes and SERS to meet the escalating demand for precise and targeted environmental surveillance [113,114,115]. This innovative approach offers unparalleled advantages in detecting a wide array of environmental pollutants, from heavy metals to organic contaminants, which are often present at trace levels and can have severe ecological and health implications.

**Figure 5:**
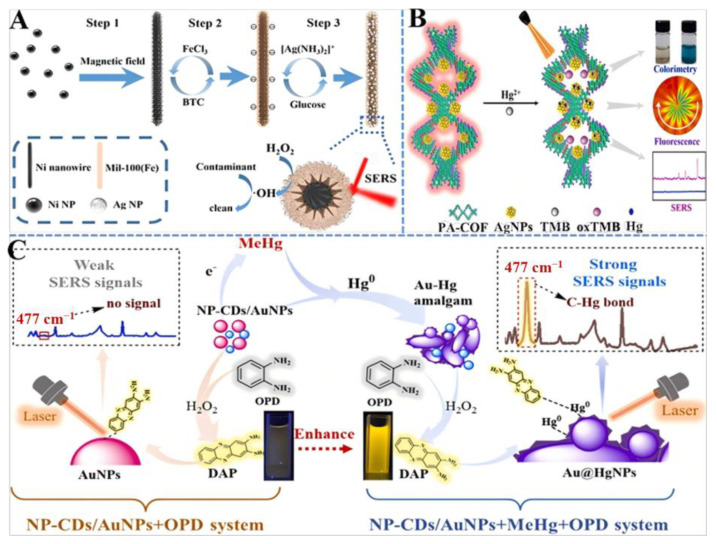
(**A**) Schematic diagram of the preparation of NMAs as a recyclable SERS probe for detection and degradation of cationic dyes [123]. (**B**) Schematic presentation of triple-readout signals platform for Hg^2+^ detection with PA-COF@AgNPs [119]. (**C**) Schematic illustration of the sensing of MeHg using Au@HgNPs [117].

As an example, Liu et al., develop a novel nanozyme-based system that not only detects but also removes organic mercury, a significant environmental pollutant [116]. The system utilizes an Au-NiFe layered double hydroxide (LDH)/rGO nanocomposite, which exhibits oxidase-like activity and acts as an efficient SERS substrate. The detection limit for MeHg detection is as low as 10^−8^ M. The mechanism involves the production of Au-amalgam on the Au surface, enhancing electron transfer and the generation of radicals, leading to the degradation of organic mercury. This study presents a nanozyme-based SERS sensor that not only detects organic mercury with high sensitivity but also enables the degradation and removal of mercury, addressing the persistence of mercury in the environment. Moreover, Li synthesizes a gold nanozyme that exhibits enhanced peroxidase-like activity in the presence of MeHg, leading to the formation of Au-Hg amalgam (Au@HgNPs) (Figure 5C) [117]. The Au@HgNPs can oxidize the weak Raman-active reporter o-phenylendiamine into the SERS-active 2,3-diaminephenazine (DAP) with a single characteristic SERS peak of C-Hg bond at 477 cm^−1^. By simulating enzyme catalytic reactions, this work enhances the detection of MeHg, revealing the versatility of nanozymes in detecting toxic metal ions.

Similarly, Zhang et al. present an efficient 2D metal–organic frameworks (MOFs) nanozyme combined with a magnetic SERS substrate for the ultrasensitive detection of Hg^2+^ [118]. The 2D MOFs nanozyme exhibits catalase-like catalytic activity, which can catalyze OPD to produce a new Raman signal. In the presence of Hg^2+^, a magnetic composite nanomaterial Fe_3_O_4_@Ag@OPD is prepared as a signal carrier, simplifying the experimental process. The method can achieve sensitive detection of Hg^2+^ with a wide detection range and a low LOD of 1.36 × 10^−13^ M, providing a new idea for detecting metal ions in water. Also, Xu et al. synthesize a fluorescent phenanthroline-functionalized covalent organic framework (PA-COF) with favorable pore structure and reactive nitrogen-containing functional groups [119]. Ag NPs are grown in PA-COF to produce a PA-COF-based nanocomposite (PA-COF@AgNPs). The formed silver amalgam in the PA-COF@AgNPs structure with the presence of Hg^2+^ can oxidize colorless TMB to a blue product (oxTMB) quickly, inducing obvious absorbance/SERS signal enhancement and fluorescence quenching of PA-COF. A triple-readout strategy is developed for highly sensitive and accurate detection of Hg^2+^, offering a promising strategy for monitoring hazardous pollutants based on the targeted fabrication of novel COFs with specific functions.

Based on bifunctional negatively charged gold nanoparticles, Xu et al. propose a rapid, simple, and sensitive surface-enhanced resonance Raman scattering sensor for the determination of hexavalent chromium (Cr(VI)) (Figure 5B) [120]. The sensor effectively promotes the conversion of TMB into oxidized TMB (oxTMB) in the presence of Cr(VI), generating a strong SERS signal at 1611 cm^−1^. The sensor exhibits a linear relationship with the logarithm of the Cr(VI) concentration from 10^−5^ to 10^−9^ M with a low limit of detection (LOD) of 0.4 nM, offering a promising analytical method for monitoring Cr(VI) in the environment. Tang et al. describe the development of Au NPs/GeO_2_ nanozymes with enhanced peroxidase-like activity, which are utilized as SERS substrates for the detection of choline iodide (ChI) [121]. The nanozymes exhibit a positive synergistic effect, enhancing both catalytic activity and SERS signal. The presence of ChI inhibits this synergy, reducing the peroxidase-like activity and SERS signal of oxTMB, which is used as a molecular probe. This inhibition forms the basis for a label-free, highly sensitive SERS method for ChI detection, demonstrating linearity in SERS signal reduction with ChI concentration over a certain range and an ultralow LOD. The method also shows good repeatability, selectivity, and applicability to real water samples, making it a promising approach for environmental monitoring of ChI.

For the control of organic contaminants, Zhao et al. focus on gold nanorods/MOFs hybrids that exhibit photo-enhanced peroxidase-like activity and SERS performance for the degradation and detection of methylene blue (MB) [122]. The hybrids can efficiently degrade MB and provide a SERS signal, making them valuable for environmental monitoring and remediation. This research combines gold nanorods with MOFs to enhance the degradation of organic dyes, demonstrating the potential of nanozyme-based SERS sensors in environmental remediation. In addition, Jiang et al. construct a peroxidase-like MOF-coated magnetic SERS probe (NMAs) for the detection and degradation of cationic dyes (Figure 5A) [123]. The probe can detect multiple cationic dyes with high sensitivity and recyclability. The probe’s peroxidase-like catalytic reaction can eliminate cationic dyes in a short time without expensive equipment or complex processes. The magnetic NMAs can be refreshed rapidly, and the probe’s sensitivity is significantly improved with a detection limit as low as 10^−10^ M for crystal violet. Focusing on the detection and degradation of cationic dyes in pond water, it utilizes a recyclable SERS probe that enhances the detection sensitivity and recyclability of the sensor, reducing environmental impact.

The above work focuses on the development of nanozyme-based SERS sensors for the detection of environmental pollutants, specifically targeting heavy metal ions. Each study explores the synthesis and application of nanozymes with enzyme-like activities that enhance the sensitivity and selectivity of SERS signals. These sensors are designed to detect contaminants such as Hg^2+^, Cr(VI), and MeHg, which are of significant environmental and health concern due to their toxicity. The common approach involves the interaction between the nanozymes and the target pollutants, leading to a change in the SERS signal. These studies also share the goal of achieving high sensitivity and specificity in detection, rapid response times, and the ability to detect pollutants at trace levels. Furthermore, the research emphasizes the cost-effectiveness and recyclability of the nanozymes, the stability of the nanozymes under various conditions, and the potential for in-field testing and monitoring in complex environmental samples. Collectively, these studies demonstrate the promise of nanozyme-based SERS sensors in environmental analysis, showing their potential for real-world applications in detecting and quantifying hazardous pollutants with high accuracy and efficiency.

### 4.3. Biomedical Marker Identification

SERS has emerged as a powerful analytical tool in the field of biomedical research, particularly for the identification and quantification of biomarkers [123,124]. When SERS is integrated with nanozymes, Raman scattering signals can be greatly enhanced by roughened metallic surfaces, such as Au NPs or Ag NPs, to detect biomolecules with high sensitivity and specificity [125,126]. Nanozyme-SERS technology has shown promise in disease diagnosis by detecting compounds such as H_2_O_2_, uric acid (UA), glutathione (GSH), cholesterol, alpha-fetoprotein (AFP), and carcinoembryonic antigen (CEA) in serum, which are indicative of various disorders and cancers.

**Figure 6:**
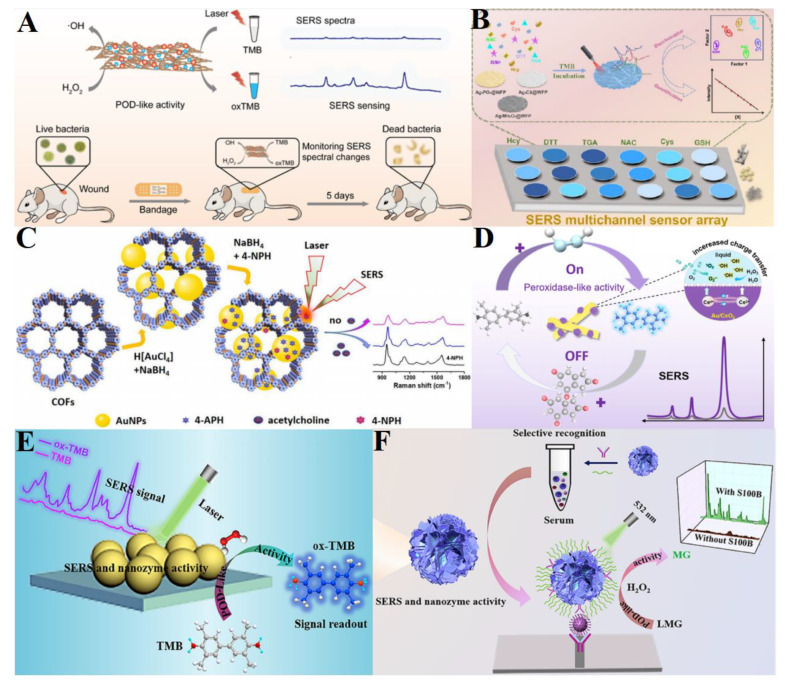
(**A**) Schematic presentation of the detection of H_2_O_2_ for antibacterial therapy with AuNP-Cu^2+^-C_3_N_4_ [129]. (**B**) Construction of SERS multichannel paper-based sensor array for analysis of biothiols [140]. (**C**) Schematic diagram of AuNPs@COF nanozyme for SERS-based acetylcholine detection [137]. (**D**) Schematic illustration of the SERS sensor for UA using Au/CeO_2_ nanozyme [131]. (**E**) Scheme of the Mo_2_N nanozyme combined with SERS for the detection of GSH, AFP, and CEA [134]. (**F**) Scheme presentation of label-free SERS detection of S100 calcium-binding protein with MoO_3_−x/CuS [142].

For example, researchers report on the development of novel SERS sensors that utilize the catalytic activity of nanozymes for the ultrasensitive detection of H_2_O_2_, a reactive oxygen species with significant implications in biological processes and various diseases [63,127,128]. The sensors are designed using different nanomaterials, such as CeO_2_@nanogel/Au, Ag/GQDs, and Mn_3_O_4_-Au nanocomposites, which mimic the activity of natural enzymes like peroxidase to catalyze the oxidation of substrates like TMB, leading to a color change and distinct Raman signal. The studies highlight the high sensitivity, selectivity, and stability of these SERS sensors, which are crucial for accurate H_2_O_2_ detection in complex biological samples, including intracellular environments, food, and environmental samples. Furthermore, these nanozyme-based SERS sensors demonstrate potential applications in monitoring H_2_O_2_ levels during cellular processes, such as apoptosis and cancer cell metabolism (Figure 6A) [129], as well as in real samples like body fluids [130]. The common theme across these studies is the innovation in functional nanomaterials that enable the precise detection of H_2_O_2_ at low concentrations, which is vital for diagnostic, environmental monitoring, and biomedical applications.

In addition, the application of nanozyme-based SERS sensors has been discussed for the detection of UA and H_2_O_2_ [131,132,133]. A shared pattern observed within these studies is the utilization of nanozymes, which mimic the activity of natural enzymes, to catalyze the oxidation of specific substrates in the presence of H_2_O_2_ or UA, leading to a detectable change in the SERS signal. These nanozymes, such as Au/CeO_2_ nanorods (Figure 6D) [131], VO-MnCo_2_O_4_/Ag [132] and silver-carbon dots (Ag-CDs) nanocomposites [62], often based on metal or metal oxide nanoparticles, exhibit peroxidase-like activity and enhance the SERS signals of the oxidized products, such as oxTMB. The studies highlight the high sensitivity, selectivity, and stability of these nanozyme-SERS sensors, which are crucial for accurate detection in complex biological samples like serum and urine. Additionally, they emphasize the simplicity and cost-effectiveness of the detection methods, as well as their potential for point-of-care testing and early diagnosis of diseases associated with abnormal levels of UA and H_2_O_2_.

In addition, nanozyme-based SERS sensors have been explored for the detection of biomolecules, specifically focusing on glutathione (GSH), alpha-fetoprotein (AFP), and carcinoembryonic antigen (CEA) (Figure 6E), and cholesterol [62,134,135]. The studies highlight the synthesis of novel nanomaterials, such as bifunctional Mo_2_N nanoparticles [134] and AgNPs@MOF [135], which exhibit both peroxidase-like activity and SERS activity. These materials are adopted to catalyze the oxidation of colorless substrates into SERS-active products, enabling the sensitive and specific detection of biomarkers (glucose, cholesterol) in complex biological samples like cells [62] and human serum [74,136]. The nanozyme-based SERS sensors have advantages such as low LODs, wide dynamic ranges, high sensitivity, and good selectivity, making them promising tools for biolomedical analysis. Based on Au@AgPt and Au NPs@COF nanozymes, SERS sensors are successfully built for the detection of acetylcholine (Figure 6C) and *Staphylococcus aureus* in serum samples [137,138,139]. Benefiting from highly active and dense “hot spot” substrate and catalytic activity, the SERS sensors will offer a promising avenue for chemical and bioassay applications.

For the detection of other biomarkers like biothiols, dopamine, and calcium-binding protein, nanozymes such as Ag-containing precipitates (Figure 6B) [140], AgNPs@PVP [141], and MoO_3_−x/CuS heterojunctions [142] (Figure 6F) are combined with SERS to enhance the detection sensitivity. These materials catalyze the oxidation of colorless substrates like TMB to produce SERS-active products, enabling the sensitive and label-free detection of targets such as biothiols and cancer cells, dopamine, and the cerebral infarction biomarker S100B. The integration of nanozymes with SERS technology offers a highly sensitive, selective, and reliable approach for monitoring important biomarkers in biological and environmental samples, with potential applications in disease diagnosis and treatment monitoring.

Nanozyme-SERS technology represents a significant advancement in the field of analytical science, offering a sensitive, specific, and versatile platform for food contaminants, environmental pollutants, biomedical markers, and beyond (Table 1). The development of such multifaceted platforms has paved the way for innovative approaches in biomedical applications, leading to the use of various nanomaterials in research practices for the sensitive detection and analysis of various biomarkers. Advances in techniques such as immunosensors for rapid detection, molecularly imprinted biosensors, microfluidics technology, and wearable biosensors have aided in the search for potential biomarkers and their characteristic “fingerprint” profiling. As research continues, this technology is expected to play an increasingly important role in clinical practices and biomedical research.

## 5. Perspectives and Challenges

Through the integration of nanozymes with SERS technology, nanozymes-based SERS sensors represent a significant leap forward in the field of analytical chemistry, particularly in food safety, environmental monitoring, and biomedical diagnostics. As we look to the future, several perspectives and challenges emerge that will shape the development and application of these sensors.

(I) Deepening understanding of nanozyme principles and mechanisms: A deeper understanding of the principles and mechanisms underlying nanozyme activity is essential. While significant progress has been made in the synthesis and application of nanozymes, the theoretical work and mechanism clarification remain limited. Future research should focus on elucidating the structure-activity relationships of nanozymes to guide their precise design for specific applications. This includes understanding how the physicochemical properties of nanozymes, such as size, morphology, and surface groups, influence their catalytic performance and selectivity.
(II) Development of standardized characterization systems: The development of standardized systems for characterizing nanozyme performance is a critical challenge. Nanozymes differ significantly from natural enzymes, and traditional characterization methods may not be directly applicable. Establishing uniform systems and standards will facilitate the comparison of different nanozymes and their catalytic activities. This is particularly important for the Michaelis-Menten kinetics, which are commonly used to discuss natural enzymes but may not fully capture the heterogeneous mechanisms of nanozymes on nanomaterial surfaces.
(III) Engineering nanozymes with tailored properties: Another significant challenge is the engineering of nanozymes with tailored properties for specific applications. As size, morphology, and surface chemistry significantly influence enzymatic activity, it is crucial to achieve high-performance nanozymes by controlling these parameters. Research should focus on developing methods to controllably engineer nanozymes and extend their functions through surface modifications, such as the introduction of functional groups or the attachment of specific recognition elements like antibodies or aptamers.
(IV) Evaluating high-performance nanozymes: The evaluation of high-performance nanozymes is essential for developing improved analytical techniques. While various nanozymes have been reported for signal production and amplification, their catalytic activity in real applications is still relatively low. There is a need for nanozymes with high catalytic activity, diverse enzymatic activities, and good substrate selectivity. This challenge requires the development of new materials and synthetic strategies to create nanozymes that can catalyze specific substrates efficiently.
(V) Integrating diverse techniques: The integration of distinct techniques with nanozymes to create multi-modal detection platforms is a promising area for future research. Combining nanozymes with techniques such as molecular imprinting, fluorescence, and electrochemistry can enhance the detection specificity, selectivity, and sensitivity. This integration can lead to the development of next-generation analytical tools that are more powerful and versatile than current methods.
(VI) Addressing real-world complexity: A significant challenge in the application of nanozyme-based SERS sensors is addressing the complexity of real-world samples. These sensors must be able to selectively detect target analytes in the presence of a multitude of interfering substances. Research should focus on improving the selectivity and robustness of nanozyme-SERS sensors to ensure accurate detection in complex matrices.
(VII) Scaling up and commercialization: Finally, the challenge of scaling up the production of nanozymes and their integration into SERS sensors for commercial use cannot be overlooked. This involves not only the development of cost-effective and large-scale synthesis methods but also the standardization of sensor fabrication and performance. Commercialization will require addressing issues related to sensor stability, reproducibility, and user-friendliness.

In conclusion, the future of nanozyme-based SERS sensors looks promising, with the potential to revolutionize analytical chemistry in various sectors. However, it will be crucial to address these challenges for realizing the full potential of these sensors and translating them from the laboratory to real-world applications.

## Figures and Tables

**Table 1:** Application of nanozyme-based SERS sensors in food safety, environmental and biomedical detection (Note: /, not reported).

Material	Linear Range	LOD	Detection Time (T)/ Reproducibility (RSD or CV)/Stability (S)	Target	Real Samples	Recovery (%)	Ref.
Food contaminants detection							
Ag@AuIP6	2–200 pg/L	0.58 pg/L	RSD: 8.09%	AFB1	Corn	79.49–105.09%	[93]
Cu_2_O@Au	0.001–100 ng/mL	0.7 pg/mL	RSD: 5.2% S: 10 day	AFB1	Peanut	93.6–102.3%	[94]
Ce-CDs/ AuNPs	0.125–87.5 μg/L	0.08 μg/L	/	AFB1	Peanut oil	93.97–109.30%	[95]
S-CDs/AuNPs	0.25–18.75 μg/L	0.29 μg/L	RSD: 4.88%	OTA	Coffee	96.7–108.9%	[96]
MAP@Ab	1.0–500 ng/mL	0.032 ng/mL	RSD: 2% S: 10 day	MC-LR	Dongpo Lake water	86.37–96.27%	[78]
FeMOF@OCTB	0.02–1.2 nmol/L	0.010 nmol/L	RSD: 4.5%	IPS	Rice	97.7–104%	[99]
Au@Pt	2.5 × 10^−7^–1.0 × 10^−8^ mol/L	9.23 × 10^−9^ mol/L	RSD: 4.86% S: 10 day	CAP	Milk	100.4–104.5%	[100]
Au@Apts	10^−10^–10^−1^ mol/L	2.04 × 10^−11^ mol/L	/	Tobramycin	Milk and eggs	94.4–102%	[59]
MnO_2_@AuNPs	10^1^–10^7^ CFU/mL	1.561 CFU/mL	/	S. aureus	Milk, apple juice, milk tea, water, and human serum	85–105%	[101]
Au@Pt	10–10^4^ CFU/mL	/	/	S. typhi	Milk	/	[102]
Au@Pt	1–10^6^ CFU/mL	1 CFU/mL	RSD:0.55%	Salmonella	Lake water, egg, and cabbage	/	[103]
MIL-100(Fe)@AuNPs	10^−11^–5 × 10^−3^ mol/L	3.9 × 10^−12^ mol/L	RSD: 3.7%	HA	Fermented soybean products	94.42–105.75%	[109]
Au NPs	10^−11^–10^−3^ mol/L	1.22 × 10^−12^ mol/L	RSD: 2.1%	Histamine	Fish samples and red wine	93.7–108.4%	[110]
AuNPs doped COF	25.65–6.2 × 10^4^ ng/mL	0.01 ng/mL	RSD: 6.35% S: 35 day	Allergenic proteins	Milk, yogurt, cookie, candy, PHF, and EHF	98.81–101.49%	[111]
GeO_2_ @Fe_3_O_4_/Au NPs	10^−9^–1 mol/L	6.162 × 10^−13^ mol/L	RSD: 3.1% T: 42 day	AA	Oranges, vitamin C drinks and vitamin C tablets	74.69–123.51%	[112]
Environmental pollutant detection							
NP-CDs/Au NPs	0.5–105.5 μg/L	0.12 μg/L	RSD: 3.17%	MeHg	Water samples	106.48–120.69%.	[117]
Fe_3_O_4_@Ag@OPD@S1	1.0 × 10^−12^–1.0 × 10^−2^ mol/L	1.36 × 10^−13^ mol/L	RSD: 4.72 %	Hg^2+^	River	96.8–106.5 %	[118]
PA- COF@AgNPs	0.05–100 μmol/L	2 × 10^−5^ μmol/L	/	Hg^2+^	Tap water samples	86.00–105%	[119]
AuNPs	10^−5^–10^−9^ mol/L	0.4 nmol/L	RSD: 5.12%	Cr (VI)	River water and industrial wastewater.	90.64–111.83%	[120]
Au NPs/GeO	10^−2^–10^−7^ mol/L	3.11 × 10^−10^ mol/L	RSD: 5%	ChI	Tap water	91.11–107.37%	[121]
Au NRs/Fe-MOF	10^−9^–10^−5^ mol/L	9.3 × 10^−12^ mol/L	RSD: 2.2%	MB	Tap wate	97.0–110.0%	[122]
Ni@Mil-100 (Fe) @Ag	10^−6^–10^−10^ mol/L	10^−10^ mol/L	RSD:9.27%	CV	/	/	[123]
Biomedical marker identification							
Ag/Mn_3_O_4_, Ag_3_PO_4_ and Ag_3_Cit	1–100 μmol/L	/	RSD: 1.97%	GSH	Tumor Cells	/	[140]
AuNPs@COF	0.001–10.0 nmol/L	0.3 pmol/L	RSD: 4.86% S: 30day	Ach	Serum	97.2–104.5%	[137]
Au/CeO_2_	10^−8^–10^−2^mol/L	3.29 × 10^−10^ mol/L	RSD: 0.018% S: 42 day	UA	Serum and urine	98.6–102.5%	[131]
Mo_2_N	0–100 µmol/L 0.1–1000 ng/mL 0.1–1000 ng/mL	0.1 μmol/L, 89.1, 74.6 pg/mL	RSD: 7%	GSH, AFP, and CEA	Serum	96.0–101%	[134]
MoO_3−_x/CuS	1 × 10^−6^–1 ug/mL	0.47 pg/mL	RSD:5.6% S:90 day	S100B	Serum	93.5–108%	[142]

## References

[1] Zhang L., Qi Z., Yang Y., Lu N., Tang Z. (2024). Enhanced “electronic tongue” for dental bacterial discrimination and elimination based on a DNA-encoded nanozyme sensor srray. ACS Appl. Mater. Interfaces.

[2] Wang Z., Dong M., Pan Y., Zhang L., Lei H., Zheng Y., Shi Y., Liu S., Li N., Wang Y. (2024). Turning threat to therapy: A nanozyme-patch in surgical bed for convenient tumor vaccination by sustained in situ catalysis. Adv. Healthc. Mater..

[3] Yue X., Fu L., Wu C., Xu S., Bai Y. (2023). Rapid trace detection of sulfite residue in white wine using a multichannel colorimetric nanozyme sensor. Foods.

[4] Singh R., Umapathi A., Patel G., Patra C., Malik U., Bhargava S.K., Daima H.K. (2023). Nanozyme-based pollutant sensing and environmental treatment: Trends, challenges, and perspectives. Sci. Total Environ..

[5] Shi Y., Ma Z., Zhang X., Ma Z., Yan F., Zhu C., Chen Y. (2024). Single-atom nanozyme-like lanthanum moieties for high-performance electromagnetic energy absorption. Adv. Funct. Mater..

[6] Wang Y., Yang Y., Liu J., Zi X., Zhu H., Sun X., Miao Y., Fu Y. (2024). Revolutionizing nanozyme technology with metal-organic frameworks: Classification, catalytic mechanisms, regulation and applications in biotechnology. Chem. Eng. J..

[7] Wu L., Zhou S., Wang G., Yun Y., Liu G., Zhang W. (2021). Nanozyme applications: A glimpse of insight in food safety. Front. Bioeng. Biotechnol..

[8] Bilal M., Khaliq N., Ashraf M., Hussain N., Baqar Z., Zdarta J., Jesionowski T., Iqbal H.M.N. (2023). Enzyme mimic nanomaterials as nanozymes with catalytic attributes. Colloids Surf. B..

[9] Peng F.F., Zhang Y., Gu N. (2008). Size-dependent peroxidase-like catalytic activity of Fe_3_O_4_ nanoparticles. Chin. Chem. Lett..

[10] Ju J., Chen Y., Liu Z., Huang C., Li Y., Kong D., Shen W., Tang S. (2023). Modification and application of Fe_3_O_4_ nanozymes in analytical chemistry: A review. Chin. Chem. Lett..

[11] Liu S., Liao Y., Shu R., Sun J., Zhang D., Zhang W., Wang J. (2024). Evaluation of the multidimensional enhanced lateral flow immunoassay in point-of-care nanosensors. ACS Nano.

[12] Singh S. (2019). Nanomaterials exhibiting enzyme-like properties (nanozymes): Current advances and future perspectives. Front. Chem..

[13] Ruan Y., Li Q., Yang D., Yang Y. (2024). Fluorescence detection of valence speciation of Cr(III) based on the catechol oxidase mimic enzyme activity of CuSeNP nanozymes. Microchim. Acta.

[14] Chandio I., Ai Y., Wu L., Liang Q. (2023). Recent progress in MOFs-based nanozymes for biosensing. Nano Res..

[15] Campuzano S., Pedrero M., Yáñez-Sedeño P., Pingarrón J.M. (2020). Nanozymes in electrochemical affinity biosensing. Microchim. Acta.

[16] Menbere Leul M., Ebrahim M.A., Andrea C., Wolfgang F. (2024). Frontiers in laccase nanozymes-enabled colorimetric sensing: A review. Anal. Chim. Acta..

[17] Su Y., Zhang Q., Miao X., Wen S., Yu S., Chu Y., Lu X., Jiang L.-P., Zhu J. (2019). Spatially engineered janus hybrid nanozyme toward SERS liquid biopsy at nano/microscales. ACS Appl. Mater. Interfaces.

[18] Mu M., Wen S., Hu S., Zhao B., Song W. (2022). Putting surface-enhanced Raman spectroscopy to work for nanozyme research: Methods, materials and applications. Trac Trend Anal. Chem..

[19] Kneipp K., Kneipp H., Kneipp J. (2006). Surface-enhanced Raman scattering in local optical fields of silver and gold nanoaggregatesfrom single-molecule Raman spectroscopy to ultrasensitive probing in live cells. Acc. Chem. Res..

[20] Wang Y., Wang Y., Lee L.Y.S., Wong K.Y. (2023). An emerging direction for nanozyme design: From single-atom to dual-atomic-site catalysts. Nanoscale.

[21] Waris N., Hasnat A., Hasan S., Bano S., Sultana S., Ibhadon A.O., Khan M.Z. (2023). Development of nanozyme based sensors as diagnostic tools in clinic applications: A review. J. Mater. Chem. B.

[22] Zhang W., Taheri-Ledari R., Saeidirad M., Qazi F.S., Kashtiaray A., Ganjali F., Tian Y., Maleki A. (2022). Regulation of porosity in MOFs: A review on tunable scaffolds and related effects and advances in different applications. J. Environ. Chem. Eng..

[23] Mu Z., Wang Y., Guo J., Zhao M. (2024). Active site-tuned high peroxidase-like activity nanozyme for on-the-spot detection of saliva total antioxidant capacity using smartphone devices. Talanta.

[24] Kulandaivel S., Lin C.-H., Yeh Y.-C. (2021). The bi-metallic MOF-919 (Fe–Cu) nanozyme capable of bifunctional enzyme-mimicking catalytic activity. Chem. Comm..

[25] Tong P.H., Wang J.J., Hu X.L., James T.D., He X.P. (2023). Metal–organic framework (MOF) hybridized gold nanoparticles as a bifunctional nanozyme for glucose sensing. Chem. Sci..

[26] Jiang B., Liang M. (2020). Advances in single-atom nanozymes research. Chin. J. Chem..

[27] Li K., Miao Y., Song K., He S., Zhang G., Waterhouse G.I.N., Guan S., Zhou S. (2023). Collaborative CuMn diatomic nanozyme to boost nanocatalytic/mild photothermal/chemo-therapy through overcoming therapeutic resistance. Chem. Eng. J..

[28] Yan X., Liu N., Liu W., Zeng J., Liu C., Chen S., Yang Y., Gui X., Yang G., Yu D. (2024). Recent advances on COF-based single-atom and dual-atom sites for oxygen catalysis. Chem. Comm..

[29] Pudlarz A.M., Ranoszek-Soliwoda K., Czechowska E., Tomaszewska E., Celichowski G., Grobelny J., Szemraj J. (2018). A Study of the activity of recombinant Mn-superoxide dismutase in the presence of gold and silver nanoparticles. Appl. Biochem. Biotechnol..

[30] Nath A., Pal R., Singh L.M., Saikia H., Rahaman H., Ghosh S.K., Mazumder R., Sengupta M. (2018). Gold-manganese oxide nanocomposite suppresses hypoxia and augments pro-inflammatory cytokines in tumor associated macrophages. Int. Immunopharmacol..

[31] Mazrad Z.A.I., Lee K., Chae A., In I., Lee H., Park S.Y. (2018). Progress in internal/external stimuli responsive fluorescent carbon nanoparticles for theranostic and sensing applications. J. Mater. Chem. B.

[32] Gusain R., Singhal N., Singh R., Kumar U., Khatri O.P. (2016). Ionic-liquid-functionalized copper oxide nanorods for photocatalytic splitting of water. ChemPlusChem.

[33] Ovejero Paredes K., Díaz-García D., García-Almodóvar V., Lozano Chamizo L., Marciello M., Díaz-Sánchez M., Prashar S., Gómez-Ruiz S., Filice M. (2020). Multifunctional silica-based nanoparticles with controlled release of organotin metallodrug for targeted theranosis of breast cancer. Cancers.

[34] Lin X.J., Sun T.Q., Yang L.P., Sun Y.G., Bin D.S., Duan S.Y., Liu Y., Lv R.W., Cao A.M. (2018). A facile synthetic strategy for the creation of hollow noble metal/transition metal oxide nanocomposites. Chem. Commun..

[35] Liu B., Liu J. (2017). Surface modification of nanozymes. Nano Res..

[36] Maddinedi S.B., Mandal B.K., Anna K.K. (2016). Environment friendly approach for size controllable synthesis of biocompatible Silver nanoparticles using diastase. Environ. Toxicol. Pharmacol..

[37] Moreira Da Silva C., Girard A., Le Bouar Y., Fossard F., Dragoe D., Ducastelle F., Loiseau A., Huc V. (2023). Structural size effect in capped metallic nanoparticles. ACS Nano.

[38] Zhong S., Zhang Z., Zhao Q., Yue Z., Xiong C., Chen G., Wang J., Li L. (2024). Lattice expansion in ruthenium nanozymes improves catalytic activity and electro-responsiveness for boosting cancer therapy. Nat. Commun..

[39] Pham X.H., Tran V.K., Hahm E., Kim Y.H., Kim J., Kim W., Jun B.H. (2022). Synthesis of gold-platinum core-Shell nanoparticles assembled on a silica template and their peroxidase nanozyme properties. Int. J. Mol. Sci..

[40] Han X., Zheng B., Ouyang J., Wang X., Kuang Q., Jiang Y., Xie Z., Zheng L. (2012). Control of anatase TiO_2_ nanocrystals with a series of high-energy crystal facets via a fuorine-free strategy. Chem. Asian J..

[41] Hashad R.A., Singla R., Kaur Bhangu S., Jap E., Zhu H., Peleg A.Y., Blakeway L., Hagemeyer C.E., Cavalieri F., Ashokkumar M. (2022). Chemoenzymatic surface decoration of Nisin-shelled nanoemulsions: Novel targeted drug-nanocarriers for cancer applications. Ultrason. Sonochem..

[42] Jimenez Jimenez A.M., Rodrigo M.A.M., Milosavljevic V., Krizkova S., Kopel P., Heger Z., Adam V. (2016). Gold nanoparticles-modified nanomaghemite and quantum dots-based hybridization assay for detection of HPV. Sens. Actuators B Chem..

[43] Wang Y., Xianyu Y. (2022). Nanobody and nanozyme-enabled immunoassays with enhanced specificity and sensitivity. Small Methods.

[44] Wang Z., Li M., Bu H., Zia D.S., Dai P., Liu J. (2023). Nanomaterials for molecular recognition: Specific adsorption and regulation of nanozyme activities. Mater. Chem. Front..

[45] Zhang L., Wang H., Qu X. (2023). Biosystem-inspired engineering of nanozymes for biomedical applications. Adv. Mater..

[46] Hu Z., Zhou X., Zhang W., Zhang L., Li L., Gao Y., Wang C. (2024). Photothermal amplified multizyme activity for synergistic photothermal-catalytic tumor therapy. J. Colloid Interface Sci..

[47] Liu Q., Zhang A., Wang R., Zhang Q., Cui D. (2021). A review on metal-and metal oxide-based nanozymes: Properties, mechanisms, and applications. Nano-Micro Lett..

[48] Wu Y.Y., Tian X., Jiang Y., Ma H.Y., Wang W., Zhang W.S., Martin J.S., Yan Y., Qin D.-D., Han D.X. (2024). Advances in bimetallic materials and bimetallic oxide nanozymes: Synthesis, classification, catalytic mechanism and application in analytical chemistry. Trends Anal. Chem..

[49] Su L., Qin S., Xie Z., Wang L., Khan K., Tareen A.K., Li D., Zhang H. (2022). Multi-enzyme activity nanozymes for biosensing and disease treatment. Coord. Chem. Rev..

[50] Ruan H., Zhang S., Wang H., Pei J., Zhao R., Mu X., Wang H., Zhang X. (2022). Single-Atom Pd/CeO_2_ nanostructures for mimicking multienzyme activities. ACS Appl. Nano Mater..

[51] Liang M., Yan X. (2019). Nanozymes: From new concepts, mechanisms, and standards to applications. Acc. Chem. Res..

[52] Yu R.J., Li Q., Liu S.C., Ma H., Ying Y.L., Long Y.T. (2023). Simultaneous observation of the spatial and temporal dynamics of single enzymatic catalysis using a solid-state nanopore. Nanoscale.

[53] Li C., Yu Y., Li H., Lin H., Cui H., Pan Y., Zhang R., Song Y., Shum H.C. (2023). Heterogeneous self-assembly of a single type of nanoparticle modulated by skin formation. ACS Nano.

[54] Liu Y., Liu R., Ai C., Wang B., Chu R., Wang H., Shui L., Zhou F. (2022). Stick-slip-motion-assisted interfacial self-assembly of noble metal nanoparticles on tapered optical fiber surface and its application in SERS detection. Appl. Surf. Sci..

[55] Hu P., Yang H., Si R., Wei B., Wang X., Xu Z., Yang X., Guo T., Gebauer R., Teobaldi G. (2024). Atomically thin Ag nanosheets for single-molecule SERS detection of BPF. Chem.

[56] Chen Q., Wang Z., Liao H., Li R., Feng Z., Li Z., Lin L., Li J., Chen G. (2024). EM and CT synergistic SERS enhancement in Si/TiO_2_/Ag heterostructures via local interfacial effect. Chem. Eng. J..

[57] Ma N., Zhang X.Y., Fan W., Guo S., Zhang Y., Liu Y., Chen L., Jung Y.M. (2019). SERS study of Ag/FeS/4-MBA interface based on the SPR effect. Spectrochim. Acta A.

[58] Wen P., Yang F., Tang L., Hu X., Zhao H., Tang B., Chen L. (2022). Precise regulation and control of hotspots in nanoparticle multilayer SERS substrates. Microchem. J..

[59] Li P., Wang B., Qi M., Jiang H., Li Y., Zhang X. (2023). Construction of aptamer sensor based on Au nanozymes for ultrasensitive SERS detection of tobramycin. J. Food Compost. Anal..

[60] Rastogi L., Karunasagar D., Sashidhar R.B., Giri A. (2016). Peroxidase-like activity of gum kondagogu reduced/stabilized palladium nanoparticles and its analytical application for colorimetric detection of glucose in biological samples. Sensor Actuators B Chem..

[61] Wang Z., Feng L., Xiao D., Li N., Li Y., Cao D., Shi Z., Cui Z., Lu N. (2017). A silver nanoislands on silica spheres platform: Enriching trace amounts of analytes for ultrasensitive and reproducible SERS detection. Nanoscale.

[62] Li Y., Li P., Chen Y., Wu Y., Wei J. (2023). Interfacial deposition of Ag nanozyme on metal-polyphenol nanosphere for SERS detection of cellular glutathione. Biosens. Bioelectron..

[63] Siddiqui S., Niazi J.H., Qureshi A. (2021). Mn_3_O_4_-Au nanozymes as peroxidase mimic and the surface-enhanced Raman scattering nanosensor for the detection of hydrogen peroxide. Mater. Today Chem..

[64] Xia X., Weng Y., Zhang L., Tang R., Zhang X. (2021). A facile SERS strategy to detect glucose utilizing tandem enzyme activities of Au@Ag nanoparticles. Spectrochim. Acta A.

[65] Liu D., Gao H., Jiang W., Yan S., Liu H., Chen J., Wen S., Zhang W., Wang X., Zhao B. (2023). Ag aerogel-supported single-atom Hg nanozyme enables efficient SERS monitoring of enhanced oxidase-like catalysis. Anal. Chem..

[66] Wen S., Ma X., Liu H., Chen G., Wang H., Deng G., Zhang Y., Song W., Zhao B., Ozaki Y. (2020). Accurate monitoring platform for the surface catalysis of nanozyme validated by surface-enhanced Raman-kinetics model. Anal. Chem..

[67] Zeng M., Zhang C., Yao Q., Jin J., Ye T., Chen X., Guo Z., Chen X. (2024). Multifunction nanoenzyme-assisted ion-selective and oxidation catalysis SERS biosensors for point-of-care nitrite testing. Sensor Actuators B Chem..

[68] Yao D., Li C., Wang H., Wen G., Liang A., Jiang Z. (2020). A new dual-mode SERS and RRS aptasensor for detecting trace organic molecules based on gold nanocluster-doped covalent-organic framework catalyst. Sensor Actuators B Chem..

[69] Arshad F., Nurul Azian Zakaria S., Ahmed M.U. (2024). Gold-silver-platinum trimetallic nanozyme-based dual-mode immunosensor for ultrasensitive osteoprotegerin detection. Microchem. J..

[70] Bhattacharjee G., Majumder S., Senapati D., Banerjee S., Satpati B. (2020). Core-shell gold @silver hollow nanocubes for higher SERS enhancement and non-enzymatic biosensor. Mater. Chem. Phys..

[71] Damborska D., Bertok T., Dosekova E., Holazova A., Lorencova L., Kasak P., Tkac J. (2017). Nanomaterial-based biosensors for detection of prostate specific antigen. Microchim. Acta.

[72] Jeon T.Y., Kim D.J., Park S.G., Kim S.H., Kim D.H. (2016). Nanostructured plasmonic substrates for use as SERS sensors. Nano Converg..

[73] Ma W., Liu L., Zhang X., Liu X., Xu Y., Li S., Zeng M. (2022). A microfluidic-based SERS biosensor with multifunctional nanosurface immobilized nanoparticles for sensitive detection of MicroRNA. Anal. Chim. Acta.

[74] Huang Y., Gu Y., Liu X., Deng T., Dai S., Qu J., Yang G., Qu L. (2022). Reusable ring-like Fe_3_O_4_/Au nanozymes with enhanced peroxidase-like activities for colorimetric-SERS dual-mode sensing of biomolecules in human blood. Biosens. Bioelectron..

[75] Zhang J., Miao X., Song C., Chen N., Xiong J., Gan H., Ni J., Zhu Y., Cheng K., Wang L. (2022). Non-enzymatic signal amplification-powered point-of-care SERS sensor for rapid and ultra-sensitive assay of SARS-CoV-2 RNA. Biosens. Bioelectron..

[76] Lin Y., Bariya M., Nyein H.Y.Y., Kivimäki L., Uusitalo S., Jansson E., Ji W., Yuan Z., Happonen T., Liedert C. (2019). Porous enzymatic membrane for nanotextured glucose sweat sensors with high stability toward reliable noninvasive health monitoring. Adv. Funct. Mater..

[77] Cottat M., D’Andrea C., Yasukuni R., Malashikhina N., Grinyte R., Lidgi-Guigui N., Fazio B., Sutton A., Oudar O., Charnaux N. (2015). High sensitivity, high selectivity SERS detection of MnSOD using optical nanoantennas functionalized with aptamers. J. Phys. Chem. C.

[78] Wu L., Jiao L., Xue D., Li Y., Han Y., Wei O., Chen Q. (2024). Nanozyme and bifunctional nanobody-based colorimetric-SERS dual-mode Immunosensor for microcystin-LR detection. Food Chem..

[79] Yu H., Wang M., Cao J., She Y., Zhu Y., Ye J., Wang J., Lao S., Abd El-Aty A.M. (2021). Determination of dichlorvos in pears by surface-enhanced Raman scattering (SERS) with catalysis by platinum coated gold nanoparticles. Anal. Lett..

[80] Zhang X., Wu D., Zhou X., Yu Y., Liu J., Hu N., Wang H., Li G., Wu Y. (2019). Recent progress on the construction of nanozymes-based biosensors and their applications to food safety assay. Trac Trend Anal. Chem..

[81] Li Y., Mu Z., Yuan Y., Zhou J., Bai L., Qing M. (2023). An enzymatic activity regulation-based clusterzyme sensor array for high-throughput identification of heavy metal ions. J. Hazard. Mater..

[82] Zhao X., Niu R., Fan S., Jing X., Gao R., Yang H., Wang H., Wang D., Yang Z., Xie Y. (2022). A dual-mode NADH biosensor based on gold nanostars decorated CoFe_2_ metal–organic frameworks to reveal dynamics of cell metabolism. ACS Sens..

[83] Tang X., Wen R., Ji C., Wei J., Han Y., Wu L. (2024). Electrochemical potential enhanced EC-SERS sensor for sensitive and label-free detection of acetamiprid. Microchem. J..

[84] Liu C., Wu T., Zeng W., Liu J., Hu B., Wu L. (2022). Dual-signal electrochemical aptasensor involving hybridization chain reaction amplification for aflatoxin B1 detection. Sens. Actuators B Chem..

[85] Long W., Shuhong Z., Yonghuan Y., Lin Z., Bei L., Weimin Z. (2021). A multifunctional probe for lead(II) sensing using CdSe/ZnS-luminol-conjugated Fe3O4 magnetic nanocomposites. Sens. Actuators B Chem..

[86] Wu L., Tang X., Wu T., Zeng W., Zhu X., Hu B., Zhang S. (2023). A review on current progress of Raman-based techniques in food safety: From normal Raman spectroscopy to SESORS. Food Res. Int..

[87] Liu J., Hong Z., Yang W., Liu C., Lu Z., Wu L., Foda M.F., Yang Z., Han H., Zhao Y. (2021). Bacteria inspired internal standard SERS substrate for quantitative detection. ACS Appl. Bio Mater..

[88] Logan N., Cao C., Freitag S., Haughey S.A., Krska R., Elliott C.T. (2024). Advancing mycotoxin detection in food and feed: Novel insights from surface-enhanced Raman spectroscopy (SERS). Adv. Mater..

[89] Zhang W., Peng Y., Lin C., Xu M., Zhao S., Li D., Yang Y., Yang Y. (2024). A novel ultra-sensitive semiconductor SERS substrate V5S4 nanopompons for the specific detection of antibiotics with AI technology. Chem. Eng. J..

[90] Wu T., Tang X., Zeng W., Han Y., Zhang S., Wei J., Wu L. (2024). Potential powered EC-SERS for sensitive detection of acetamiprid. J. Food Eng..

[91] Xia J., Li W., Sun M., Wang H. (2022). Application of SERS in the detection of Fungi, Bacteria and Viruses. Nanomaterials.

[92] Guo Z., Chen P., Yosri N., Chen Q., Elseedi H.R., Zou X., Yang H. (2023). Detection of heavy metals in food and agricultural products by surface-enhanced Raman spectroscopy. Food Rev. Int..

[93] Tan X., Kang K., Zhang R., Dong J., Wang W., Kang W. (2024). An ultrasensitive dual-mode approach for AFB1 detection: Colorimetric/label-free SERS aptasensor based on a self-assembled core-shell structured Ag@Au IP6 bifunctional nanozyme. Sens. Actuators B Chem..

[94] Chen P., Li S., Jiang C., Wang Z., Ma X. (2023). A surface-enhanced Raman scattering aptasensor for output-signal detection of aflatoxin B1 based on peroxidase-like Cu_2_O@Au hybrid nanozyme. Food Biosci..

[95] Zhao X., Li Q., Li H., Wang Y., Xiao F., Yang D., Xia Q., Yang Y. (2023). SERS detection of Hg^2+^ and aflatoxin B1 through on-off strategy of oxidase-like Au@HgNPs/carbon dots. Food Chem..

[96] Li Q., Han Q., Yang D., Li K., Wang Y., Chen D., Yang Y., Li H. (2024). Methylmercury-sensitized “turn on” SERS-active peroxidase-like activity of carbon dots/Au NPs nanozyme for selective detection of ochratoxin A in coffee. Food Chem..

[97] Li M., Wang H., Yu X., Jia X., Zhu C., Liu J., Zhang F., Chen Z., Yan M., Yang Q. (2022). A sensitive and simple competitive nanozyme-linked apta-sorbent assay for the dual-mode detection of ochratoxin A. Analyst.

[98] Ma J., Feng G., Ying Y., Shao Y., She Y., Zheng L., Abd Ei-Aty A.M., Wang J. (2021). Sensitive SERS assay for glyphosate based on the prevention of l-cysteine inhibition of a Au-Pt nanozyme. Analyst.

[99] Li C., Yu F., Yang J., Bai H., Ma X., Jiang Z. (2023). SERS- and absorbance-based catalytic assay for determination of isocarbophos using aptamer-modified FeMOF nanozyme and in situ generated silver nanoparticles. Microchim. Acta.

[100] Zhang R., Xie S., Yang J., Zhang L., Xiong R., Sun H., Zhang H., Jiang M., He Y. (2024). A dual-mode colorimetric and surface-enhanced Raman strategy for chloramphenicol detection based on the Au@Pt nanozyme and EXPAR. Microchem. J..

[101] Dai J., Li J., Jiao Y., Yang X., Yang D., Zhong Z., Li H., Yang Y. (2024). Colorimetric-SERS dual-mode aptasensor for Staphylococcus aureus based on MnO_2_@AuNPs oxidase-like activity. Food Chem..

[102] Li Z., Hu J., Zhan Y., Shao Z., Gao M., Yao Q., Li Z., Sun S., Wang L. (2023). Coupling bifunctional nanozyme-mediated catalytic signal amplification and label-free SERS with immunoassays for ultrasensitive detection of pathogens in milk samples. Anal. Chem..

[103] Jiang H., Chang W., Zhu X., Liu G., Liu K., Chen W., Wang H., Qin P. (2024). Development of a colorimetric and SERS dual-signal platform via dCas9-mediated chain assembly of bifunctional Au@Pt nanozymes for ultrasensitive and robust salmonella assay. Anal. Chem..

[104] Gao X., Li C., He R., Zhang Y., Wang B., Zhang Z.H., Ho C.T. (2023). Research advances on biogenic amines in traditional fermented foods: Emphasis on formation mechanism, detection and control methods. Food Chem..

[105] Zhang S., Zhang N., Wang S., Li Z., Sun W., Zhou M., Zhang Y., Ma J., Wu L. (2023). Turn on fluorescent detection of biogenic amines in fish based on MnO_2_-coated and rhodamine 6G-loaded mesoporous silica nanospheres. Microchem. J..

[106] Patel P., Komorowski A.S., Mack D.P. (2023). An allergist’s approach to food poisoning. Ann. Allergy Asthma Immunol..

[107] Ferrante M.C., Mercogliano R. (2023). Focus on histamine production during cheese manufacture and processing: A review. Food Chem..

[108] Liu Y., Li H., Wang C., Chen S., Lian R., Wang W., Fu L., Wang Y. (2024). Immunological disturbance effect of exogenous histamine towards key immune cells. Food Sci. Hum. Wellness.

[109] Ma X., Xu S., Pan Y., Jiang C., Wang Z. (2024). Construction of SERS output-signal aptasensor using MOF/noble metal nanoparticles based nanozyme for sensitive histamine detection. Food Chem..

[110] Wang B., Jiang H., Tang R., Tan Y., Xia X., Zhang X. (2023). Construction of histamine aptamer sensor based on Au NPs nanozyme for ultrasensitive SERS detection of histamine. J. Food Compost. Anal..

[111] Su Y., Wu D., Chen J., Chen G., Hu N., Wang H., Wang P., Han H., Li G., Wut Y. (2019). Ratiometric surface enhanced Raman scattering immunosorbent assay of allergenic proteins via covalent organic framework composite material based nanozyme tag triggered Raman signal “Turn-on” and amplification. Anal. Chem..

[112] Qi M., Wang B., Jiang H., Li Y., Li P., Zhang X., Han L. (2024). Smartphone readable colorimetry and surface-enhanced Raman scattering (SERS) dual-mode sensing platform for ascorbic acid detection based on GeO_2_ composite nanozymes. J. Food Compos. Anal..

[113] Diao Q., Chen X., Tang Z., Li S., Tian Q., Bu Z., Liu H., Liu J., Niu X. (2024). Nanozymes: Powerful catalytic materials for environmental pollutant detection and degradation. Environ. Sci. Nano.

[114] Wang K., Meng X., Yan X., Fan K. (2024). Nanozyme-based point-of-care testing: Revolutionizing environmental pollutant detection with high efficiency and low cost. Nano Today.

[115] Sun Z., Ji X., Lu S., Du J. (2024). Shining a light on environmental science: Recent advances in SERS technology for rapid detection of persistent toxic substances. J. Environ. Sci..

[116] Liu H., Guo Y., Wang Y., Zhang H., Ma X., Wen S., Jin J., Song W., Zhao B., Ozaki Y. (2021). A nanozyme-based enhanced system for total removal of organic mercury and SERS sensing. J. Hazard. Mater..

[117] Li Q., Li K., Liu J., Chen D., Yang D., Yang Y., Li H. (2023). Simulated enzyme catalytic strategy for surface-enhanced Raman scattering detection of methylmercury by using carbon dots/gold-based nanozyme. J. Environ. Chem. Eng..

[118] Zhang R., Yang J., Cao Y., Zhang Q., Xie C., Xiong W., Luo X., He Y. (2024). Efficient 2D MOFs nanozyme combining with magnetic SERS substrate for ultrasensitive detection of Hg^2+^. Spectrochim. Acta. A.

[119] Xu Y., Wu C., Chu N., Yang J., Lin Y., Chen X. (2022). Colorimetric/fluorescent/SERS triple-readout sensing of Mercury(II) via in-situ growth of AgNPs in phenanthroline-functionalized covalent organic framework. Sens. Actuators B Chem..

[120] Xu G., Guo N., Zhang Q., Wang T., Song P., Xia L. (2022). A sensitive surface-enhanced resonance Raman scattering sensor with bifunctional negatively charged gold nanoparticles for the determination of Cr(VI). Sci. Total Environ..

[121] Tang R., Xia X., Zhang X., Jiang H., Wang B., Zhang P., Zhang Y., Tang Y., Zhou Y. (2022). Synergistic function of Au NPs/GeO_2_ nanozymes with enhanced peroxidase-like activity and SERS effect to detect choline iodide. Spectrochim. Acta A.

[122] Zhao X., Yang T., Wang D., Zhang N., Yang H., Jing X., Niu R., Yang Z., Xie Y., Meng L. (2022). Gold nanorods/metal-organic framework hybrids: Photo enhanced peroxidase-like activity and SERS performance for organic dyestuff degradation and detection. Anal. Chem..

[123] Jiang G., Wang Z., Zong S., Yang K., Zhu K., Cui Y. (2021). Peroxidase-like recyclable SERS probe for the detection and elimination of cationic dyes in pond water. J. Hazard. Mater..

[124] Hassanain W.A., Izake E.L., Sivanesan A., Ayoko G.A. (2017). Towards interference free HPLC-SERS for the trace analysis of drug metabolites in biological fluids. J. Pharm. Biomed. Anal..

[125] Cialla-May D., Bonifacio A., Bocklitz T., Markin A., Markina N., Fornasaro S., Dwivedi A., Dib T., Farnesi E., Liu C. (2024). Biomedical SERS—The current state and future trends. Chem. Soc. Rev..

[126] Vazquez-Iglesias L., Casagrande G.M.S., Garcia-Lojo D., Leal L.F., Ngo T.A., Perez-Juste J., Reis R.M., Kant K., Pastoriza-Santos I. (2024). SERS sensing for cancer biomarker: Approaches and directions. Bioact. Mater..

[127] Li Y., Zhang Y., Jiang H., Qi M., Zhang X., Zhu B., Han L. (2023). CeO_2_@nanogel/Au nanozymes to enhance peroxidase activity for a novel ultrasensitive SERS assay of H_2_O_2_ determination. Microchem. J..

[128] Jin J., Song W., Wang J., Li L., Tian Y., Zhu S., Zhang Y., Xu S., Yang B., Zhao B. (2022). A highly sensitive SERS platform based on small-sized Ag/GQDs nanozyme for intracellular analysis. Chem. Eng. J..

[129] Qu L., Han J., Huang Y., Yang G., Liu W., Long Z., Gu Y., Zhang Q., Gao M., Dong X. (2023). Peroxidase-like nanozymes for point-of-care SERS sensing and wound healing. ACS Appl. Nano Mater..

[130] Cao X., Jiang H., Huang X., Sun D., Qi G. (2025). Hydrogel patch doped with nanoenzyme for SERS detection of hydrogen peroxide in complex body fluids. Talanta.

[131] Li Y., Li P., Qi M., Zhang X., Meng J. (2024). SERS-based non-enzymatic detection of uric acid using Au/CeO_2_ nanorods. ACS Appl. Nano Mater..

[132] Tan Y., Qi M., Jiang H., Wang B., Zhang X. (2023). Determination of uric acid in serum by SERS system based on V_O_-MnCo_2_O_4_/Ag nanozyme. Anal. Chim. Acta.

[133] Wang A., Guan C., Shan G., Chen Y., Wang C., Liu Y. (2019). A nanocomposite prepared from silver nanoparticles and carbon dots with peroxidase mimicking activity for colorimetric and SERS-based determination of uric acid. Microchim. Acta.

[134] Chen Y., Zhang J., Li J., Hu Y., Ge K., Li G., Liu S. (2024). Bifunctional Mo_2_N nanoparticles with nanozyme and SERS activity: A versatile platform for sensitive detection of biomarkers in serum samples. Anal. Chem..

[135] Wu Y., Chen J.-Y., He W.-M. (2022). Surface-enhanced Raman spectroscopy biosensor based on silver nanoparticles@metal-organic frameworks with peroxidase-mimicking activities for ultrasensitive monitoring of blood cholesterol. Sens. Actuators B Chem..

[136] Xu J., Jian X., Guo J., Zhao J., Tang J., Zhao Y., Xu J., Gao Z., Song Y.-Y. (2023). Selective SERS identification and quantification of glucose enantiomers on homochiral MOFs based enzyme-free nanoreactors. Chem. Eng. J..

[137] Fu C., Li Y., Lei X., Su J., Chen Y., Wu Y., Shi W., Tan X., Li Y., Jung Y.M. (2024). SERS sensor for acetylcholine detection based on covalent organic framework hybridized gold nanoparticles as nanozymes. Anal. Chem..

[138] Huang X., Yang Y., Zhou H., Hu L., Yang A., Jin H., Zheng B., Pi J., Xu J., Sun P. (2024). Coupling of an Au@AgPt nanozyme array with an micrococcal nuclease-specific responsiveness strategy for colorimetric/SERS sensing of Staphylococcus aureus in patients with sepsis. J. Pharm. Anal..

[139] Cai J., Lin Y., Yu X., Yang Y., Hu Y., Gao L., Xiao H., Du J., Wang H., Zhong X. (2023). Multifunctional AuAg-doping Prussian Blue-based MOF: Enhanced colorimetric catalytic activities and amplified SERS signals for bacteria discrimination and detection. Sens. Actuators B Chem..

[140] Wang L., Chen Y., Ji Y., Wang L., Liu X., Wang F., Li C. (2024). Nanozyme-inhibited SERS multichannel paper-based sensor array for the quantification and identification of biothiols and cancer cells based on three Ag-based nanomaterials. Anal. Chem..

[141] Zhao L., Du X., Xu G., Song P. (2024). Nanozyme catalyzed-SERRS sensor for the recognition of dopamine based on AgNPs@PVP with oxidase-like activity. Spectrochim. Acta A.

[142] Chen Y., Zhang J., Li Y., Li G., Hu Y. (2024). Bifunctional MoO_3_–x/CuS heterojunction nanozyme-driven “Turn-On” SERS signal for the sensitive detection of cerebral infarction biomarker S100B. Anal. Chem..

